# Comparative Assessment of the Structural Features of Originator Recombinant Human Follitropin Alfa Versus Recombinant Human Follitropin Alfa Biosimilar Preparations Approved in Non-European Regions

**DOI:** 10.3390/ijms23126762

**Published:** 2022-06-17

**Authors:** Lucio Manzi, Nunzio Sepe, Walter Migliaccio, Ludovica Lanzoni, Luisa Iozzino, Fabrizia D’Angelo, Lucia Colarusso, Susana Montenegro, Angelo Palmese, Thomas D’Hooghe, Alfredo Ulloa-Aguirre, Yulia Koloda, Monica Lispi

**Affiliations:** 1 Characterization & Innovative Analytics Unit—Analytical Development Biotech—Global Analytical Development—Global Development & Launch—Global Healthcare Operation, Merck Serono S.p.A., 00176 Rome, Italy, an affiliate of Merck KGaA; lucio.manzi@merckgroup.com (L.M.); nunzio.sepe@merckgroup.com (N.S.); walter.migliaccio@merckgroup.com (W.M.); ludovica.lanzoni@merckgroup.com (L.L.); luisa.iozzino@merckgroup.com (L.I.); fabrizia.dangelo@merckgroup.com (F.D.); lucia.colarusso@merckgroup.com (L.C.); 2 Merck Healthcare KGaA, 64293 Darmstadt, Germany; susana.montenegro@merckgroup.com (S.M.); monica.lispi@merckgroup.com (M.L.); 3 Department of Development and Regeneration, Laboratory of Endometrium, Endometriosis & Reproductive Medicine, KU Leuven, Herestraat 49-Box 805, B-3000 Leuven, Belgium; 4 Department of Obstetrics, Gynecology, and Reproductive Sciences, Yale University Medical School, New Haven, CT 06510, USA; 5 Research Support Network (RAI), Universidad Nacional Autónoma de México-Instituto Nacional de Ciencias Médicas y Nutrición SZ, Tlalpan, Mexico City 14000, Mexico; aulloaa@unam.mx; 6 Department of Obstetrics and Gynecology, Russian Medical Academy of Continuous Professional Education, Centre of Reproduction “Life Line”, 121471 Moscow, Russia; julkol@yandex.ru; 7 PhD School of Clinical and Experimental Medicine, Unit of Endocrinology, University of Modena and Reggio Emilia, 41121 Modena, Italy

**Keywords:** r-hFSH-alfa, biosimilars, glycosylation, sialylation, macroheterogeneity, microheterogeneity, *N*-glycolylneuraminic acid, Neu5Gc, *O*-acetylation, post-translational modifications

## Abstract

Although the full primary structures of the alfa and beta subunits of reference r-hFSH-alfa and its biosimilars are identical, cell context-dependent differences in the expressing cell lines and manufacturing process can lead to variations in glycosylation profiles. In the present study, we compared the structural features of reference r-hFSH-alfa with those of five biosimilar preparations approved in different global regions outside Europe (Primapur^®^, Jin Sai Heng^®^, Follitrope^®^, Folisurge^®^, and Corneumon^®^) with respect to glycosylation, macro- and microheterogeneity, and other post-translational modifications and higher order structure. The mean proportion of *N*-glycosylation-site occupancy was highest in reference r-hFSH-alfa, decreasing sequentially in Primapur, Jin Sai Heng, Corneumon, Follisurge and Follitrope, respectively. The level of antennarity showed slightly higher complexity in Corneumon, Primapur and Follitrope versus reference r-hFSH-alfa, whereas Jin Sai Heng and Folisurge were aligned with reference r-hFSH-alfa across all *N*-glycosylation sites. Sialylation level was higher in Corneumon and Follitrope, but small differences were detected in other biosimilar preparations compared with reference r-hFSH-alfa. Jin Sai Heng showed higher levels of *N*-glyconeuramic acid than the other preparations. Minor differences in oxidation levels were seen among the different products. Therefore, in summary, we identified var ious differences in *N*-glycosylation occupancy, antennarity, sialylation and oxidation between reference r-hFSH-alfa and the biosimilar preparations analyzed.

## 1. Introduction

Follicle-stimulating hormone (FSH) is a pituitary gonadotropin that plays an important role in sexual development and function [1,2]. Exogenous preparations of FSH have long been used in the treatment of infertility, and can either be derived from purified human urine (u-FSH and human menopausal gonadotropin (hMG)) or produced as a recombinant product (r-hFSH), which is defined as a protein produced using DNA technology that utilizes biological processes to produce large-molecule drugs that cannot be synthesized using synthetic chemistry [3,4].

Reference r-hFSH-alfa (follitropin alfa; GONAL-f^®^, Merck Healthcare KGaA, Darmstadt, Germany (hereafter referred to as reference r-hFSH-alfa)) was the first r-hFSH-alfa preparation produced using recombinant DNA technology, with marketing authorization granted in the European Union in 1995 [3,4]. Reference r-hFSH-alfa is indicated to treat ovulatory disorders (including polycystic ovarian syndrome (PCOS)) and for the stimulation of multifollicular development in women undergoing assisted reproductive technology (ART). It is also effective in stimulating spermatogenesis, with concomitant human chorionic gonadotropin (hCG) therapy, in adult men who have congenital or acquired hypogonadotropic hypogonadism [5,6]. With a predicted 20,737,203 treatment cycles in women between the product’s international birthday of 20 October 1995 and 19 October 2021 (calculated from sales data and expected average use [7]) and using a conservative estimate of live birth rate (LBR) of 25% for reference r-hFSH-alfa (based on the randomized controlled trial by Bosch et al. [8]), more than 5 million babies are estimated to have been born following treatment with reference r-hFSH-alfa.

A biosimilar medicine (biosimilar) is a biologic medicine that is highly similar to a biologic medicine (reference product) and has been approved by the corresponding authorities, based on the regulatory definitions for biosimilars and related approval comparability guidelines in the respective countries [9,10]. Following the expiration of the compound patent for reference r-hFSH-alfa in many European countries, marketing authorization was granted in Europe for two r-hFSH-alfa biosimilar medicines, in line with European Medicines Agency (EMA) guidelines: Ovaleap^®^ (Theramex, Dublin, Ireland [11]) and Bemfola^®^ (Gedeon Richter, Budapest, Hungary [12]). To obtain regulatory approval from the EMA, r-hFSH-alfa biosimilars must show comparable structural, physico-chemical, pharmacological, pharmacokinetic, toxicological, efficacy and safety profiles to reference r-hFSH-alfa, which are assessed in comparability studies [13]. In the wider global context, the decision to approve biosimilars is generally based on a full comparability exercise, including data from comprehensive quality, non-clinical and clinical studies, conducted by the local regulatory body [10].

The International Conference on Harmonisation (ICH) Guidance for Industry, Comparability of Biotechnological/Biological Products Subject to Changes in Their Manufacturing Process (ICH Q5E), describes the scientific principles for the comparability assessment of biopharmaceuticals [14]. Demonstrating that a proposed product is a biosimilar to a reference product is a complex process, as the manufacturer of a proposed biosimilar product is likely to have applied a different manufacturing process (e.g., different cell line, raw materials, equipment, processes, process controls, and acceptance criteria) from that of the reference product, and to have no direct knowledge of the manufacturing process for the reference product. Furthermore, extensive characterization of the physico-chemical (e.g., composition and physical properties), structural (primary structure, higher-order structure, and post-translational modifications, such as glycosylation), biological, pharmacological and immunochemical properties, purity, and impurities, plays a crucial role in determining that a product is a biosimilar. However, despite these efforts to harmonize the regulatory framework for biosimilars globally, the adoption of biosimilar guidance and the subsequent approval of biosimilars still varies considerably between countries [15].

While the full primary structures of the alfa and beta subunits of reference r-hFSH-alfa and its biosimilars are in general identical, differences in the expressing cell lines (e.g., a distinct cell context) and manufacturing process can lead to variations in product quality, including differences in the glycosylation profiles. In 2015, a review of all glycosylated biosimilars approved in the European Union and Japan (including Bemfola and Ovaleap, epoetin alfa, epoetin kappa, epoetin zeta and infliximab) highlighted structural variations between biosimilars and their reference products [16]. The r-hFSH-alfa biosimilar Ovaleap displayed a slight shift in α2-3 sialic acid content and an increase in non-human sialic acid variants containing *N*-glyconeuramic acid [16]. The r-hFSH-alfa biosimilar Bemfola exhibited a higher proportion of tri- and tetra-antennary structures, a lower number of bi-antennary structures and variations in the proportion of α-fucosylated complexes; however, the O-acetyl-containing moieties of the α-subunit were below the level of detection [16]. Compared with reference r-hFSH-alfa, Bemfola also showed a higher level of monosialo-tri-antennary glycans and higher batch-to-batch variability [4,17], and Ovaleap showed higher total sialic acid content [18].

Oocyte number is the recommended primary outcome stipulated by the EMA for biosimilars containing r-hFSH, whereby equivalent efficacy between the test product and the reference product should be demonstrated and equivalence margins prospectively defined and justified [19]. Accordingly, Bemfola and Ovaleap were approved in the EU based on Phase III clinical trials demonstrating non-inferiority to r-hFSH-alfa for the number of oocytes retrieved and comparable safety [20,21]. However, live birth is increasingly being recognized as the standard clinical end-target measurement to estimate the success of infertility treatments [22,23,24], particularly considering the potential effects of different FSH preparations with distinct glycosylation patterns on oocyte quality [25,26]. Although the Phase III trials used for marketing authorization of these biosimilars also reported on live birth outcomes as well as clinical and ongoing pregnancy rates, these were not the primary endpoints of the studies and, consequently, their corresponding statistical analyses were not sufficiently powered to detect differences in these outcomes [27]. A recent meta-analysis, which included data from five unique randomized controlled trials evaluating the efficacy and safety of biosimilar r-hFSH-alfa preparations compared with reference r-hFSH-alfa, demonstrated a lower probability of live birth and pregnancy (ongoing and clinical) in couples treated with the biosimilar preparations compared with reference r-hFSH-alfa [28]. This meta-analysis also showed that the biosimilar preparations carried a similar risk of OHSS, ectopic pregnancy and multiple pregnancy compared with reference r-hFSH [28].

**Figure 1:**
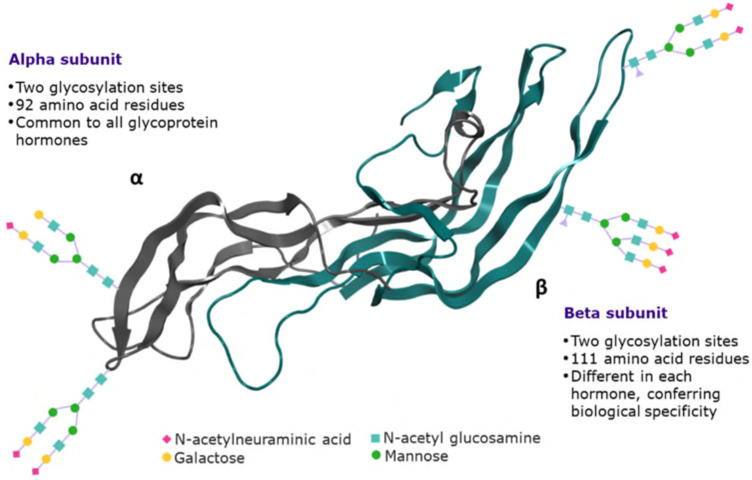
Graphical representation of human follicle-stimulating hormone.

The aim of the present study was to compare the structural features of reference r-hFSH-alfa (Figure 1) with those of five biosimilar preparations, with respect to macroheterogeneity (*N*-glycosylation site occupancy), microheterogeneity (antennarity and terminal sialylation of the oligosaccharides attached to the protein core), *N*-glycolylneuraminic acid (NGNA) content and *O*-acetylation, other post-translational modifications (oxidation, conversion of asparagine to succinimide, and N-terminal heterogeneity) and higher-order structure (extrinsic fluorescence spectroscopy profiles) (Box 1). The biosimilar preparations tested were Primapur^®^ (follitropin alfa; iVFarma, LLC, Moscow, Russia), Jin Sai Heng^®^ (follitropin alfa; GeneScience Pharmaceuticals Co., Ltd., Changchun, China), Follitrope^®^ (follitropin alfa; LG Life Sciences, Seoul, Korea), Folisurge^®^ (follitropin alfa; Intas Pharmaceuticals Ltd., Ahmedabad, India) and Corneumon^®^ (follitropin alfa; Laboratorios Corne, SA de CV, Escobedo, Mexico) (Table 1). These compounds were included in the analysis because they are the main biosimilars in regions outside of Europe (e.g., Russia and the Asia–Pacific and Latin American regions). Furthermore, their authorization was granted based on the regulatory definitions for biosimilars and related approval comparability guidelines in their respective countries, which may have different regulatory definitions and requirements for biosimilars and related approval comparability guidelines, including non-clinical and clinical aspects [10].

FSH is a heterodimeric glycoprotein composed of two non-covalently linked protein subunits: the alfa subunit (which is common to all glycoprotein hormones) and a unique beta subunit (which confers biological specificity) [29]. Each subunit contains two *N*-glycosylation sites (α subunit: Asn 52 and Asn 78; β subunit: Asn 7 and Asn 24; Figure 1), which occur at the asparagine (Asn, N) amino acid when it is positioned in a glycosylation sequon (Asn-X-Ser/Thr, where X can be any amino acid followed by either serine or threonine) [30]. In the beta subunit, the number of occupied *N*-glycosylation sites varies (zero, one or two), whereas in the alfa subunit both *N*-glycosylation sites are occupied. Accordingly, four hFSH glycoforms have been identified, based on glycan occupancy: hFSH24, which possesses *N*-glycans at all four sites; hFSH21, which lacks the βAsn24 glycan; hFSH18, which lacks the βAsn7 glycan; and hFSH15, which lacks both FSHβ N-glycans. The two most abundant human FSH glycoforms are FSH24 and FSH21 [31].

Box 1 **Explanation of terms.**

**Macroheterogeneity** relates to the presence or absence of a oligosaccharide chain at any of the two known *N*-glycosylation sites (site occupancy) [17] in the beta subunit. In vivo, the macroheterogeneity of circulating hFSH is dynamic and may have a physiologic role. In women of reproductive age, more glycosylated (fully glycosylated) and acidic glycoforms (with prolonged in vivo half-life due to reduced renal clearance) are secreted during the early and mid-follicular phases, compared with less sialylated, glycosylated (hypo-glycosylated) glycoforms, which are more predominant before ovulation [32,33]. Highly acidic isoforms are more predominant after the menopause than during the fertile lifespan [34]. Furthermore, tri-glycosylated hFSH (hFSH18/21) is more abundant in young women, whereas tetra- glycosylated (hFSH24) and highly sialylated forms are more abundant in peri/postmenopausal women [32,35]. Glycosylation at αAsn 52 is essental for FSH bioactivity, as it has an important role in the assembly of the functional FSH heterodimer and its subsequent stability; it also has a pivotal role in FSH receptor (FSHR) activation and signalling, whereby glycoforms with smaller and more compact glycosylation at αAsn 52 can fit into the central cavity of the FSHR more rapidly than bulkier and extended glycans, leading to a more rapid response [17].**Microheterogeneity** relates to the structural variation in the type of carbohydrates comprising the oligosaccharide chains attached to the protein core and the branching of these chains (antennarity) into bi-, tri- and tetra-antennary structures [36,37,38]. The building block *N*-acetyl glucosamine (GlcNAc) is linked to asparagine followed by the addition of another GlcNAc, then by one to three Mannose residues that can branch into 1 to 4 antennae. The antennae are then extended by GlcNAc and galactose, the latter of which can be capped by *N*-acetyl neuraminic acid (sialic acid) [30]. The average number of antennae per glycan is reflected by the A-Index, which is calculated for each *N*-glycosylation site and as a mean over the entire molecule (see Section 4).**Sialylation** relates to the inclusion of sialic acid in the glycoprotein antennae. The addition of a sialic acid cap imparts a negative charge on each antenna [30]. The degree of sialic acid capping can vary among glycoforms, whereby variants with a high degree of sialylation are more acidic than those with low sialic acid content [36,37,38]. A high level of sialylation, in combination with the presence of bulkier glycans, may contribute to a longer half-life through reduced glomerular filtration and, therefore, higher net in vivo potency. CHO cells, in which r-hFSH is produced, do not have the ability to synthesize sialic acid attached in the position α2-6, so only α2-3 sialic acid is found in reference r-hFSH-alfa and biosimilar preparations [30]. The average number of sialic acid moieties per glycan is reflected by the S-Index, which is calculated for each *N*-glycosylation site and as a mean over the entire molecule (see Section 4). The NGNA index reflects the content of glycans with *N*-glyconeuramic acid (NGNA) moieties. NGNA may be linked to immunogenic reactions, as humans do not produce CMP-*N*-acetylneuraminic acid hydroxylase, the enzyme responsible for this glycan modification [38]. Anti-NGNA activity has been reported in 85% of healthy humans, suggesting its potential for eliciting an immune response in humans [39]. The NGNA index is calculated for each *N*-glycosylation site and as a mean over the entire molecule (see Section 4).***O*-acetylation:** The nine-carbon backbone of sialic acids can undergo extensive enzymatic modification in nature, and *O*-acetylation at the C-4/7/8/9 positions in particular is widely observed [40]. *O*-acetylation increases the hydrophobic character of sialylated glycans and can change the biophysical properties of the glycoprotein, potentially leading to changes in activity and glycan antigen recognition [40].**Post-translational modifications** can include the oxidation of methionine residues. The methionine residues in FSH are not directly located in regions that are critical for binding to the FSHR: methionine 29 is involved in α–β subunit heterodimerization; methionine 47 is located close to the FSHR binding site, but is not directly involved in ligand–receptor interaction; methionine 71 is located close to the heterodimerization site but is not directly involved in heterodimerization; and methionine 109 is located in the non-structural C-terminal region. However, the oxidation of these residues may lead to conformational changes, with the potential for indirect effects on biological activity, pharmacokinetics or protein aggregation, and the alteration of the immunogenicity of therapeutic proteins [41]. In contrast to the post-translational modifications in the oligosaccharide chains attached to the protein core, other modifications, such as the conversion of asparagine to succinimide and N-terminal clipping, are not known to have an impact on biological activity.**Higher-order structure** refers to the self-assembly into either the seconday-, tertiary- and quarternary-order structure of a protein. Higher-order structure is responsible for the correct folding and three-dimensional shape of a protein and is strongly dependent on the protein environment; therefore, different formulations can bear conformational differences compared with the reference preparation, which can have an impact on the activity of the molecule.

## 2. Results

### 2.1. Macroheterogeneity (Degree of N-Glycosylation Site Occupancy)

**Figure 2:**
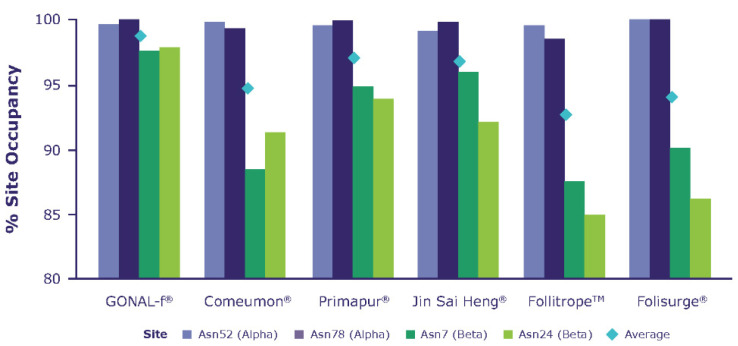
Proportion of *N*-glycosylation site occupancy, measured as an average of all tested batches per biosimilar preparation.

Peptide mapping combined with the use of *N*-glycosidases allows the pinpointing of the location of the *N*-glycosylation sites of each subunit of the molecule and the calculation of the fraction linked to an oligosaccharide chain (*N*-glycosylation site occupancy). Figure 2 shows the average proportion of *N*-glycosylation site occupancy, measured as the mean of all tested batches per biosimilar preparation. As expected, the results show differences in the site occupancy of the ß-subunit; specifically, reference r-hFSH-alfa showed higher site occupancy versus the other preparations tested, mainly in the β subunit.

The bar chart reports the average *N*-glycosylation sites (bars) calculated over all the batches listed in Table 1. The diamonds represent the average occupancy calculated over all glycosylation sites. A clear decreasing trend in *N*-glycosylation sites is observed when comparing the reference r-hFSH-alfa (GONAL-f) versus biosimilar preparations.

### 2.2. Microheterogeneity (Degree of Antennarity, Sialylation, NGNA, and O-Acetylation)

**Figure 3:**
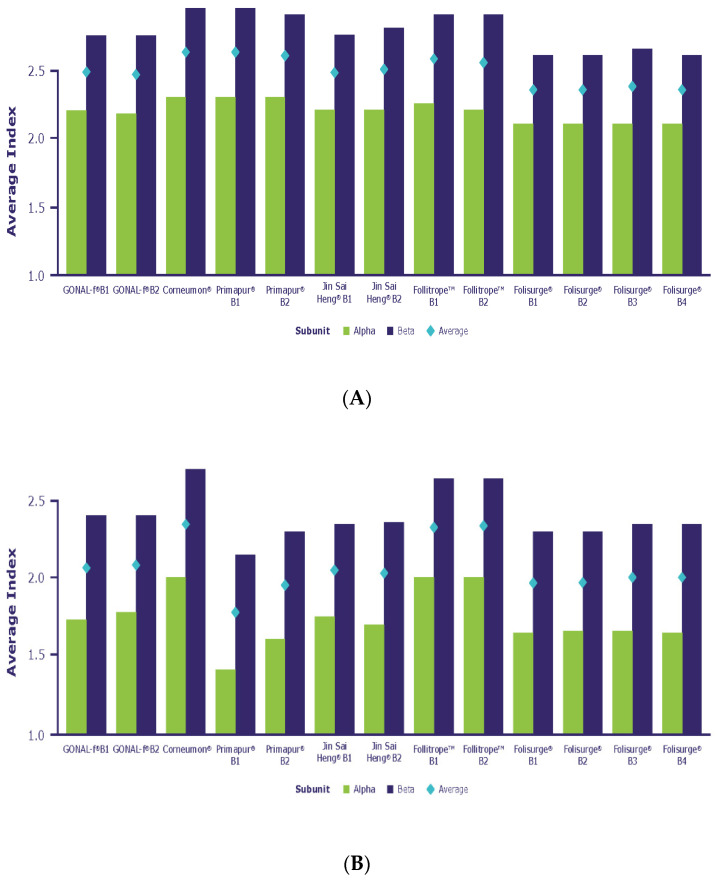
(**A**) Antennarity (A-Index) and (**B**) sialylation (S-Index) of selected biosimilar preparations.

Glycopeptide mapping allows the determination of the site-specific distribution of glycan structures across the various *N*-glycosylation sites. The following section describes the glycan structural features observed on each glycosylation site of the biosimilar FSH compounds analyzed in this study. The data are grouped according to the features of each glycan that reflect their degree of branching (antennarity), sialylation, NGNA and *O*-acetylation. The results are detailed in Figure 3 and in Table 2 and Table 3.

The extent of glycan branching and the degree of sialylation are key features of glycan structure. The average number of antennae and sialic acid moieties per glycan is reflected by the A-Index and the S-Index, respectively. Such indexes are calculated for each *N*-glycosylation site and as a mean over the entire molecule.

#### 2.2.1. Antennarity

The extent of glycan branching is a key feature of glycan structure. The average number of antennae per glycan is reflected by the A-Index, which was calculated for each *N*-glycosylation site and as a mean over the entire molecule. Figure 3 shows that Corneumon, Primapur and Follitrope showed an average distribution of glycan antennarity that was slightly skewed towards higher values when compared with reference r-hFSH-alfa, indicating a slightly higher level of bulkier glycans in these preparations. These minor discrepancies were consistent in both subunits. Jin Sai Heng and Folisurge were aligned with reference r-hFSH-alfa across all *N*-glycosylation sites.

#### 2.2.2. Sialylation

The S-Index averaged across all *N*-glycosylation sites revealed a higher degree of sialylation in Corneumon and Follitrope (2.4 and 2.3, respectively) compared with that of reference r-hFSH-alfa (S-Index 2.1). Jin Sai Heng and Folisurge had a similar degree of sialylation to reference r-hFSH-alfa (2.0 for both preparations) and Primapur had a lower degree of sialylation (1.85) (Supplementary Table S1). In all cases, there was considerable variation in the S-Index for both the alfa and the beta subunits among the preparations tested. For example, compared with reference r-hFSH-alfa, the S-index was higher for the beta subunit of Corneumon, lower for the alfa subunit of Primapur, and higher for both the alfa and beta subunits of Follitrope (Figure 3; Supplementary Table S1).

#### 2.2.3. NGNA Content and *O*-Acetylation

Most biosimilars showed a lower content of NGNA when compared with reference r-hFSH-alfa. Jin Sai Heng, on the other hand, showed a higher level of NGNA-containing glycans (Table 2 and Table 3). These results are consistent for all *N*-glycosylation sites in which NGNA-containing glycans were detected. *O*-Acetylation is a common modification of sialic acid moieties that can be monitored using various analytical techniques. The con- tent of acetylated sialic acid in reference r-hFSH-alfa was higher than that observed in Primapur and Follitrope, whereas Folisurge and Jin Sai Heng showed over 8-fold and 10-fold increases, respectively, in sialic acid acetylation when compared with reference r-hFSH-alfa (Table 2 and Table 3).

### 2.3. Post-Translational Modifications

**Figure 4:**
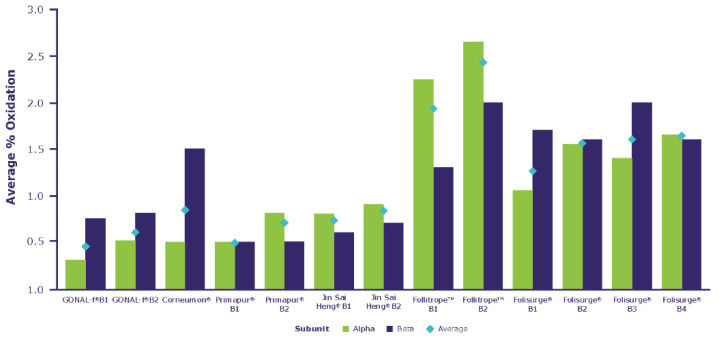
Comparative analysis of oxidation levels in the preparations.

As shown in Supplementary Table S2, the post-translational modifications identified included the oxidation of methionine, the conversion of asparagine to succinimide and N-terminal clipping. The conversion of asparagine to succinimide and the clipping of N-terminal residues, not including αCys7 and βCys3, are not known to have an impact in terms of the efficacy or safety of the product. There were some minor differences in the level of oxidation across the different products. As shown in Figure 4, Corneumon, Jin Sai Heng, and Primapur were aligned in terms of average oxidation levels compared with the reference r-hFSH-alfa, whereas Folisurge and Follitrope showed a slightly higher oxidation level, distributed across these biosimilars. While we cannot rule out the possibility of oxidation due to storage, stability studies have shown that the oxidation levels of r-hFSH-alfa do not increase with appropriate storage and handling [42,43], although no data were available for the other preparations tested.

### 2.4. Higher-Order Structure

**Figure 5:**
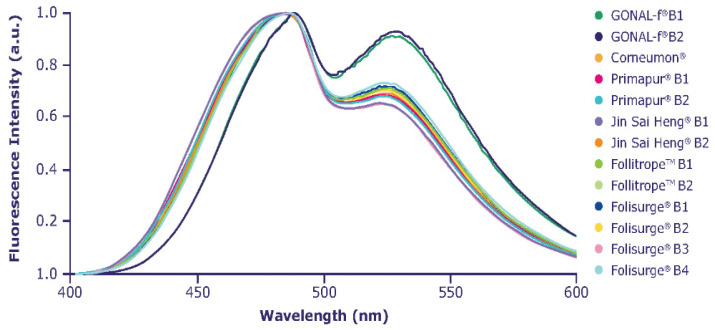
Extrinsic fluorescence spectroscopy profile.

ANS normalized emission spectra are shown in Figure 5. A different ratio among the intensity of the first and second emission bands with respect to reference r-hFSH-alfa was clearly visible for all biosimilars, indicating different interactions with ANS. This phenomenon is possibly due to a higher exposure of hydrophobic regions in the biosimilar compounds. The more pronounced differences were detected for Jin Sai Heng and Primapur, where a shift in the λmax of emission towards lower wavelengths was observed.

## 3. Discussion

As required by the local regulatory authorities, r-hFSH-alfa biosimilars must undergo full non-clinical and clinical assessments to demonstrate comparable structural, physicochemical, pharmacological, pharmacokinetic, toxicological, efficacy and safety profiles to the reference r-hFSH-alfa [15,19]. It has been acknowledged that r-hFSH-alfa biosimilars are not exact copies of reference r-hFSH-alfa [27], and differences in the glycosylation profiles between reference r-hFSH-alfa and r-hFSH-alfa biosimilars have been extensively reported [4,16,17,18], although such differences do not form the basis of the definition of biosimilars, as they are not generic drugs. The differences are, how- ever, relevant, as the biological function and action of r-hFSH in the gonads is finely regulated by glycan structures, which are also critical factors in determining the circulatory half-life of the gonadotropin [30].

The biosimilar preparations analyzed herein showed differences in glycosylation profiles when compared with reference r-hFSH-alfa. The mean proportion of *N*-glycosylation-site occupancy (i.e., the presence or absence of Asn-linked oligosaccharides at a particular site) was highest in reference r-hFSH-alfa, decreasing sequentially in Primapur, Jin Sai Heng, Corneumon, Folisurge and Follitrope, respectively (Figure 2). The greatest differences in the proportion of site occupancy between preparations was seen at βAsn 24 and βAsn 7.

The level of antennarity (i.e., the degree of branching of the oligosaccharides), as indicated by the A-index, showed the minor enrichment of bulkier glycans in Corneumon, Primapur, and Follitrope compared with reference r-hFSH-alfa. These differences, albeit minor, were consistently detected on both subunits. Jin Sai Heng and Folisurge were aligned with reference r-hFSH-alfa across all *N*-glycosylation sites. Relatively smaller FSH glycans, such as the bi-antennary FSH isoform at αAsn 52 in naturally occurring FSH and in reference r-hFSH-alfa, can better fit into the central cavity of the FSH receptor (FSHR), while more bulky/complex glycoforms (e.g., tri- and tetra-antennary FSH isoforms at αAsn 52) likely require more time to fit into the central cavity due to steric hindrance, potentially resulting in a delayed response [44]. Although glycosylation-site occupancy on the alfa and beta subunits is not directly involved in FSH binding to the FSHR because of their orientation in the heterodimer [45], macroheterogeneity in the beta subunit may indirectly influence the dynamics of the ligand–receptor interaction [36].

The level of sialylation was clearly higher in Corneumon and Follitrope, but small differences in sialylation levels were detected in other biosimilar preparations when compared with reference r-hFSH-alfa. These differences are reflected by the different S-Index values calculated for the preparations (Figure 3). The degree of terminal sialylation determines the circulatory half-life [30], whereby more acidic glycoforms (i.e., those with greater sialylation) have a longer plasma half-life due to slower clearance by the liver [30,36] than less acidic glycoforms. More acidic glycoforms are suggested to present less affinity for FSHR binding in animal and in vitro models [30,46]. However, studies in murine animal models must be verified clinically, because naturally occurring glycoforms of follitropin with fewer filled glycosylation sites at the beta subunit have been shown to bind to the FSHR more rapidly and are more active at the target cell level in both human granulosa cells and HEK293 cells than fully glycosylated follitropin [30].

In the present analysis, Jin Sai Heng showed higher levels of the sialic acid NGNA than the other preparations, as shown by the NGNA index, which reflects the content of glycans with NGNA moieties. NGNA may be linked to immunogenic reactions, as humans do not produce CMP-N-acetylneuraminic acid hydroxylase, the enzyme that catalyzes this glycan modification [38]. Anti-NGNA activity has been reported in 85% of healthy humans, suggestting its potential for eliciting immunogenicity in humans [39]. Furthermore, the Jin Sai Heng and Folisurge preparations had a higher degree of *O*-acetylation than the other preparations. *O*-acetylated *N*-acetylneuraminic acid is a naturally occurring post-glycosylation acetylation in humans, and thus it represents a low risk for immunogenicity.

Among the identified post-translational modifications, the level of oxidation showed some minor differences across the different products. As shown in Figure 4, Folisurge and Follitrope showed higher mean oxidation levels across the molecule compared with the other preparations, for which the oxidation levels were aligned. Previous analyses have reported that highly purified, urinary-derived gonadotropin preparations (HMG- HP and u-FSH) contain 49–51% oxidized proteins (compared with <2% for r-hFSH), which was presumed to be the result of oxidative stress due to the rigorous extraction and purification processes to which the urinary-derived gonadotropins were subjected [42,43]. Methionine residues can be readily oxidized to methionine sulfoxide. While the methionine residues in FSH are not directly located in regions that are critical for binding to the FSHR, the oxidation of methionine may lead to conformational changes in the protein, with the potential for indirect effects on biological activity, pharmacokinetics and/or protein aggregation, and the alteration of the immunogenicity of therapeutic proteins [41] (Box 1).

All biosimilars showed different extrinsic fluorescence spectroscopy emission profiles, with λmax shifted towards lower wavelength values compared with reference r-hFSH-alfa, which may possibly be due to a higher exposure of hydrophobic regions that interact with ANS. These differences could be related to conformational variability due to the different formulations/preparations, although there are no data on the potential impact of these differences in vivo.

The comparability studies previously performed for the r-hFSH-alfa biosimilar preparations [47,48,49,50,51,52,53] were compliant with the regulatory requirements in each of the regions (i.e., the number of oocytes retrieved). However, based on the results from the meta-analysis by Chua et al. [28], it should be noted that final fertility outcomes are different between reference r-hFSH-alfa and r-hFSH-alfa bio- similars. LBR was significantly lower with biosimilar preparations (Bemfola, Ovaleap and Primapur) versus the reference product [28]. A secondary analysis of the combined data for biosimilar preparations resulted in significantly lower live birth and clinical and ongoing pregnancy rates with biosimilar r-hFSH-alfa preparations compared with the reference product [28]. A significantly higher clinical pregnancy rate (*p* = 0.03) was observed with reference r-hFSH-alfa compared with bio-similar r-hFSH-alfa in another recently published meta-analysis [54]. However, no statistical differences were found for biochemical pregnancy rate, take-home baby rate, total r-hFSH-alpha dose, duration of stimulation, or OHSS risk [54].

Differences in physico-chemical properties among biosimilar preparations might influence the clinical outcomes of treatment. Indeed, several authors have hypothesized that differences in the abundance and/or composition of the glycoforms, glycosylation pat- terns and clearance rates observed among different FSH preparations may affect their interaction with the FSHR in the ovary and, therefore, have an impact on the quality of the oocytes [3,25,26,27]. In an in vitro study, 80% of mice follicles exposed to the less acidic human FSH for 3 days developed into two-cell embryos after in vitro mat- uration/in vitro fertilization, compared with only 60% of follicles exposed for 5 days to the more acidic isoforms [55]. Furthermore, in a meta-analysis eval- uating two pure FSH preparations (highly purified urinary FSH (Metrodin-HP^®^; Merck Healthcare KGaA, Germany) and reference r-hFSH-alfa), which differed in their glyco- sylation patterns, the relatively less acidic reference r-hFSH-alfa performed better than the relatively more acidic highly purified urinary FSH in terms of the number of follicles, number of oocytes retrieved, and duration of gonadotropin treatment [56].

In the clinical setting, choosing between reference r-hFSH-alfa and a biosimilar may prove challenging, and the decision whether to use reference r-FSH-alfa or a biosimilar preparation should be made according to the criteria of the treating physician, based on clinical efficacy and safety, real-world effectiveness, cost-effectiveness and patient preference [27]. The rapidly growing use and validation of large, computerized medical records and related databases (e.g., health insurance or national registries) have played a major part in changing the perceptions of observational data among researchers and clinicians and should be also taken into account [57]. For example, a non-interventional study based on the French National Health System (SNDS) database demonstrated that among 214,539 stimulations, reference r- hFSH-alfa was associated with significantly higher cumulative live birth rates compared with hMG-HP and r-hFSH-alfa biosimilars [58].

Furthermore, it is a commonly held opinion among experts that while r-hFSH-alfa biosimilars (Bemfola and Ovaleap) are highly similar to reference r-hFSH-alfa, they are not identical to the reference product and should not be used interchangeably [30]. Most countries do not currently have clear guidance on the interchangeability of biosimilars [10]. In the European Union, this decision is within the remit of the individual member states [10]. For example, in June 2021, the Norwegian Medicines Agency assessed Bemfola and GONAL-f to be unsuitable for exchange in pharmacies as the regulatory agency considered the preparations to have “significant differences in the administration equipment and have partly different areas of application” [59]. Similarly, in Australia, r-hFSH-alfa biosimilars cannot be used interchangeably with reference r-hFSH-alfa [60].

Head-to-head randomized controlled trials, as well as real-world studies, are still needed to assess the comparative clinical effectiveness (in terms of LBR and CLBR) and to better understand the potential differences in medically assisted reproductive outcomes between reference r-hFSH-alfa and its biosimilars. It should also be considered that changes to the upstream or downstream manufacturing processes for biosimilars have the potential to impact on the biosimilar itself or its bioactivity. For example, Sinegubova et al. (2022) recently described a pilot study for an updated purification process for Primapur that incorporates immunoaffinity chromatography, which yields a sufficiently pure product with a high degree of batch-to-batch consistency [61].

## 4. Materials and Methods

### 4.1. Samples

Two batches of reference r-hFSH-alfa (GONAL-f), one batch of Corneumon, two batches of Primapur, two batches of Jin Sai Heng, two batches of Follitrope, and four batches of Folisurge were available for testing. Reference r-hFSH-alfa was provided by Merck Healthcare KGaA, Darmstadt, Germany, and biosimilar batches were purchased in different markets. The different batches are detailed in Table 1.

### 4.2. Materials

Urea, 8-anilino-1-napthalensulfonic acid (ANS), iodoacetamide (IAM) and dithiothreitol (DTT) were purchased from Sigma (St. Louis, MO, USA). Trypsin Gold (mass spectrometry (MS) grade) and chymotrypsin were purchased from Promega (Madison, WI, USA). N-Glycanase was purchased from Agilent (Santa Clara, CA, USA). Tris-(hydroxyme-thyl)-aminomethane (TRIS), ethylenediaminetetraacetic acid (EDTA), trifluoroacetic acid (TFA), formic acid (FA), hydrochloric acid and liquid chromatography–mass spectrometry (LC–MS)-grade acetonitrile (ACN) were obtained from Merck Healthcare KGaA, Darmstadt, Germany. The 8M guanidine hydrochloride solution was purchased from Thermo Fisher Scientific (San Jose, CA, USA). Water used in experiments was purified with a Milli-Q system from Merck Millipore (Milford, MA, USA). Amicon Ultra 3K centrifugal filters were purchased from Merck Millipore (Watford, UK). An Acquity UPLC BEH glycan column 1.7 μm, 2.1 × 150 mm was purchased from Waters (Milford, MA, USA). A Kinetex Core Shell column 1.7 µm, EVO C18 100 Å, 100 mm × 2.1 µm was purchased from Phenomenex (Torrance, CA, USA).

### 4.3. Peptide Mapping for Assessment of Post-Translational Modifications and of Macroheterogeneity (N-Glycosylation Site Occupancy)

The identity, location and distribution of post-translational modifications were investigated using peptide mapping. The analysis of post-translational modifications is a common practice in characterization studies of biopharmaceuticals, since the presence or absence of a particular modification, or a variation in its relative abundance, may impact the bioactivity, immunogenicity, pharmacokinetics, pharmacodynamics, and safety of the molecule. For this reason, the relative abundance of selected post-translational modifications was investigated in the framework of this study in order to detect differences in the distribution of post-translational modifications between reference r-hFSH-alfa and its corresponding biosimilars.

Thirty micrograms of protein were concentrated to 1 µg/µL using Amicon Ultra 3K cartridges, and an equal volume of 8M guanidine, 100 mM TRIS and 1 mM EDTA pH 7.6 was then added. Samples were reduced by adding 3 µL of DTT 200 mM (1 h, 37 °C) and then alkylated with 8.3 µL of IAM 200 mM (1 h, room temperature, in the dark). Each sample was washed five times with 100 μL of 6 M Urea and 100 mM TRIS pH 8.0 using Amicon Ultra 3K cartridges, reaching a final volume of 100 μL. Washed samples were subjected to enzymatic hydrolysis with Trypsin Gold (enzyme to substrate ratio 1:10, 2 h, 37 °C) in the presence of 200 µL of 50 mM TRIS pH 8.0. Following hydrolysis, one 151.5 µL aliquot was acidified with 10 µL of 10% FA and stored at −20 °C, while the other 151.5 µL aliquot was further hydrolyzed with N-Glycanase (18 h, 37 °C). A total of 3 µL of each sample was analyzed by ultra-performance liquid chromatography–electrospray ionization tandem mass spectrometry (UPLC–ESI–MS/MS) on a Synapt G2-Si Q-TOF mass spectrometer in MSE mode, equipped with an Acquity UPLC system (Waters, Milford, MA, USA). Peptides were eluted at a flow rate of 400 µL/min, keeping the column at 60 °C, with a mixture of 0.1% FA in water (Solution A) and 0.1% FA in ACN (Solution B), employing a gradient starting at 99% Solution A. Initial conditions were held for 2 min followed by a 50 min gradient in which the proportion of Solution B was increased to 40%. The column was then washed for 3 min with 80% Solution B and then re-equilibrated for 10 min under the initial conditions. The mass spectrometer was operated with the following parameters: capillary voltage 3 kV, sampling cone 30 V, source temperature 100 °C, desolvation temperature 300 °C, cone gas flow 20 L/H, desolvation gas flow 800 L/H, and scan range 50–2000 m/z. The data were processed using Waters Biopharma Lynx 1.3.4 software (Milford, MA, USA). The identification of peptides and related post-translational modifications were performed using a 50 ppm mass accuracy threshold followed by manual inspection of the mass spectra, if needed.

### 4.4. Glycopeptide Mapping for Assessment of Microheterogeneity (Indices for Antennarity, Sialylation, Acetylation and NGNA (N-Glycolyl Neuraminic Acid))

One-hundred micrograms of protein were concentrated to 1 µg/µL using Amicon Ultra 3K cartridges. Then, an equal volume of 8M Guanidine, 130 mM TRIS and 1 mM EDTA pH 7.6 was added. Samples were reduced by adding 20 µL of DTT 500 mM (1 h, 37 °C) and then alkylated with 40 µL of IAM 500 mM (30 min in the dark). Each sample was washed five times with 200 μL of 2 M Urea and 50 mM TRIS pH 8.0 using Amicon Ultra 3K cartridges and then subjected to enzymatic hydrolysis with 10 µL of chymotrypsin (E/S 1:20, 4 h, 37 °C). Samples were analyzed by UPLC–ESI–MS/MS on a Waters Xevo G2-XS Q-TOF mass spectrometer in MSE mode, equipped with an Acquity UPLC system (Waters, Milford, MA, USA), by injecting 25 µL using the autodilution mode starting from 10 µL of each sample in each vial. Peptides were eluted at a flow rate of 0.2 mL/min, keeping the column at 50 °C, with a mixture of 0.1% TFA in water (Solution A) and 0.1% TFA in ACN (Solution B), employing a gradient starting at 90% Solution B. Initial conditions were held for 3 min before the proportion of Solution B was decreased to 70% and held for 5 min, followed by a 60 min gradient in which the proportion of Solution B was decreased to 45%. The column was then washed for 4 min with 20% Solution B and then re-equilibrated for 18 min under initial conditions. The mass spectrometer was operated with the following parameters: capillary voltage 3 kV, sampling cone 30 V, extraction cone 4 V, source temperature 120 °C, desolvation temperature 300 °C, cone gas flow 50 L/H, desolvation gas flow 800 L/H, and scan range 100–2500 m/z. The data were processed using Expressionist software 13.0 (Genedata, Basel, Switzerland). The identification of N-glycans was performed on a single peptide per *N*-glycosylation site using a 50 ppm mass accuracy threshold followed by manual inspection of the mass spectra. Extracted ion chromatograms (XICs) were used to calculate the relative abundance of each glycan per site. The data were then grouped according to the features of each glycan species and glycosylation indices were produced using the following equation:

*y − Index = LRel. Abundance_n_ × j_n_*

*n*

where *y* is the feature investigated (A-Index for antennarity, S-Index for sialylation, acetyl index for acetylation and NGNA index for N-glycolyl neuraminic acid), n represents each identified glycan and *j* is the number of each specific feature exhibited by the glycan (i.e., abundance of A3G3S2 is multiplied by 3 for the A-Index and by 2 for the S-Index). For the A-Index and S-Index the values obtained from the equation are divided by 100.

### 4.5. Fluorescence Spectroscopy for the Assessment of Higher-Order Glycoprotein Structure

Changes in protein conformation can be detected by fluorescence spectroscopy using the extrinsic probe 8-anilino-1-naphthalenesulfonic acid (ANS). In a polar environment, the ANS intrinsic fluorescence is extremely low, whereas in the presence of proteins the intrinsic fluorescence is enhanced by the non-covalent interaction of the probe with hydrophobic pockets in the protein molecule. The ANS signal will be shifted toward lower wavelengths proportionally to the degree of the interaction between the protein and ANS. If any conformational change occurs, the exposure of hydrophobic protein could be modified, thus affecting the interaction with the probe [62].

Mean extrinsic fluorescence was measured with the Spectro fluorimeter FP-8300 (Jasco, Tokyo, Japan), with samples diluted in water for injection to a final concentration of approximately 0.1 mg/mL. All samples were incubated in the dark for 90 min with ANS 10 mM in a molar ratio of protein to ANS of 1:100. Samples were analyzed across a wavelength range of 400–600 nm using a data interval of 1 nm. The excitation wavelength was 380 nm, with both an excitation and emission bandwidth of 5 nm. The optical path was 1 cm, and the scan speed was 500 nm/min with a response of 50 msec and medium sensitivity.

## 5. Conclusions

In this paper, we summarized differences in the glycosylation profiles between the r-hFSH-alfa biosimilars analyzed and the reference product. These comprise differences in *N*-glycosylation site occupancy, antennarity (albeit minor), sialylation and NGNA content and post-translational modifications, as well as potential conformational variability. Such differences may be therapeutically relevant, as the biological function and action of FSH in the gonads is finely regulated by glycan structures, which are also critical factors in determining the plasma half-life of the gonadotropin and the biological effect at the target cell level, thereby affecting clinical efficacy. Head-to-head randomized controlled trials, as well as real-world evidence studies, are now needed to assess the comparative clinical effectiveness and to better identify the potential differences in medically assisted reproductive outcomes between reference r-hFSH-alfa and its biosimilars.

## Figures and Tables

**Table 1:** Batches tested per preparation.

Product	Country/Company of Origin	Identifier	Batch Number *
GONAL-f	European Union; Merck Healthcare KGaA	B1	BA057111
		B2	BA064607
Primapur	Russia; iVFarma, LLC, Russia	B1	0011019A102
		B2	0010220B02
Jin Sai Heng	China; Genescience Pharmaceuticals Co., Ltd.	B1	201812049
		B2	055201910044
Corneumon	Mexico; Laboratorios Corne, SA de CV	-	RFU19002
Follitrope	South Korea; LG Life Sciences	B1	RFV19009
		B2	RFV199005
Folisurge	India; Intas Pharmaceuticals Ltd.	B1	7070091
		B2	7150015
		B3	7150014
		B4	7070093

* Based on batch availability at the time of testing.

**Table 2:** Degree of NGNA and *O*-acetylation for GONAL-f^®^, Corneumon^®^, Primapur^®^ and Jin Sai Heng^®^.

Glycosylation Site	Index	GONAL-f^®^		Corneumon^®^	Primapur^®^		Jin Sai Heng^®^	
		BA057111	BA064607	RFU19002	0011019A102	0010220B02	201812049	55201910044
Asn52-α	NGNA index	0.8	0.9	0.1	0.2	0.9	4.5	4.0
	Acetyl index	1.9	1.6	1.6	1.7	1.0	35.0	33.2
Asn78-α	NGNA index	1.0	1.2	0.2	0.3	1.0	5.5	4.6
	Acetyl index	3.9	3.5	3.6	4.0	2.6	44.7	43.0
Asn7-β	NGNA index	0.0	0.0	0.0	0.0	0.0	0.0	0.0
	Acetyl index	2.7	2.3	1.7	1.7	1.2	28.3	29.1
Asn24-β	NGNA index	0.5	0.7	0.1	0.2	0.7	3.1	2.8
	Acetyl index	5.4	4.7	3.1	5.3	2.8	58.0	57.8
Average	NGNA index	0.6	0.7	0.1	0.2	0.6	3.3	2.8
	Acetyl index	3.5	3.0	2.5	3.2	1.9	41.5	40.8

NGNA, *N*-glycolyl neuraminic acid. The NGNA index and acetyl index reflect the abundance of sialic acid modified by an *N*-glycolyl moiety or a *O*-acetyl group, respectively. The indices are calculated as the weighted sum of the values obtained by multiplying the relative abundance of each glycoform per the number of modifications exhibited by the sialic acid. These metrics do not reflect absolute values of modification but are valuable to compare the extent of modification across different samples.

**Table 3:** Degree of NGNA and *O*-acetylation for GONAL-f^®^, Follitrope^®^ and Folisurge^®^.

Glycosylation Site		Index	GONAL-f^®^		Follitrope^®^		Folisurge^®^			
			BA057111	BA064607	Korea	Korea	India	India	India	India
					RFV19009	RFV19005	7070091	7150015	7150014	7070093
Asn52-α	NGNA index		0.6	0.7	0.1	0.1	0.1	0.1	0.1	0.1
	Acetyl index		2.0	1.5	1.2	1.3	6.0	5.7	4.6	4.9
Asn78-α	NGNA index		0.9	1.1	0.2	0.2	0.3	0.3	0.3	0.3
	Acetyl index		2.7	2.1	2.4	2.4	7.6	7.5	6.0	6.4
Asn7-β	NGNA index		0.0	0.0	0.0	0.0	0.0	0.0	0.0	0.0
	Acetyl index		2.5	1.9	1.3	1.3	8.3	8.4	7.5	7.6
Asn24-β	NGNA index		0.4	0.4	0.1	0.1	0.2	0.2	0.2	0.2
	Acetyl index		5.6	4.2	3.1	3.0	16.1	15.4	13.7	14.4
Average	NGNA index		0.5	0.5	0.1	0.1	0.1	0.1	0.1	0.2
	Acetyl index		3.2	2.4	2.0	2.0	9.5	9.2	8.0	8.3

NGNA, *N*-glycolyl neuraminic acid. The NGNA index and acetyl index reflect the abundance of sialic acid modified by an *N*-glycolyl moiety or a *O*-acetyl group, respectively. The indices are calculated as the weighted sum of the values obtained by multiplying the relative abundance of each glycoform per the number of modifications exhibited by the sialic acid. These metrics do not reflect absolute values of modification but are valuable to compare the extent of modification across different samples.

## Data Availability

Any requests for data by qualified scientific and medical researchers for legitimate research purposes will be subject to Merck KGaA’s Data Sharing Policy. All requests should be submitted in writing to Merck KGaA’s data sharing portal https://www.merckgroup.com/en/research/our-approach-to-research-and-development/healthcare/clinical-trials/commitment-responsible-data-sharing.html (accessed on 14 June 2022). When Merck KGaA has a co-research, co-development, co-marketing or co-promotion agreement, or when the product has been out-licensed, the responsibility for disclosure might be dependent on the agreement between parties. Under these circumstances, Merck KGaA will endeavor to gain agreement to share data in response to requests.

## Supplementary Materials

The following supporting information can be downloaded at: https://www.mdpi.com/article/10.3390/ijms23126762/s1.

## References

[1] Marques P., Skorupskaite K., Rozario K., Anderson R., George J., Feingold K.R., Anawalt B., Boyce A., Chrousos G., de Herder W.W., Dhatariya K., Dungan K., Hershman J.M., Hofland J., Kalra S. (2022). Physiology of GnRH and Gonadotropin Secretion. Endotext.

[2] McGee E., Hsueh A. (2000). Initial and cyclic recruitment of ovarian follicles. Endocr. Rev..

[3] Bergandi L., Canosa S., Carosso A.R., Paschero C., Gennarelli G., Silvagno F., Benedetto C., Revelli A. (2020). Human Recombinant FSH and Its Biosimilars: Clinical Efficacy, Safety, and Cost-Effectiveness in Controlled Ovarian Stimulation for In Vitro Fertilization. Pharmaceuticals.

[4] Lunenfeld B., Bilger W., Longobardi S., Alam V., D’Hooghe T., Sunkara S.K. (2019). The Development of Gonadotropins for Clinical Use in the Treatment of Infertility. Front. Endocrinol..

[5] European Medicines Agency GONAL-f® Summary of Product Characteristics. https://www.ema.europa.eu/en/documents/product-information/gonal-f-epar-product-information_en.pdf.

[6] Ulloa-Aguirre A., Lira-Albarrán S. (2016). Clinical Applications of Gonadotropins in the Male. Prog. Mol. Biol. Transl. Sci..

[7] Velthuis E., Hubbard J., Longobardi S., D’Hooghe T. (2021). The Frequency of Ovarian Hyperstimulation Syndrome and Thromboembolism with Originator Recombinant Human Follitropin Alfa (GONAL-f) for Medically Assisted Reproduction: A Systematic Review. Adv. Ther..

[8] Bosch E., Havelock J., Martin F.S., Rasmussen B.B., Klein B.M., Mannaerts B., Arce J.C., ESTHER-2 Study Group (2019). Follitropin delta in repeated ovarian stimulation for IVF: A controlled, assessor-blind Phase 3 safety trial. Reprod. BioMed. Online.

[9] European Medicines Agency Biosimilars in the EU: Information Guide for Healthcare Professionals. https://www.ema.europa.eu/en/documents/leaflet/biosimilars-eu-information-guide-healthcare-professionals_en.pdf.

[10] Kang H.N., Thorpe R., Knezevic I., Casas Levano M., Chilufya M.B., Chirachanakul P., Chua H.M., Dalili D., Foo F., Gao K. (2021). Regulatory challenges with biosimilars: An update from 20 countries. Ann. N. Y. Acad. Sci..

[11] European Medicines Agency European Public Assessment Report (EPAR): Ovaleap® (Follitropin Alfa). https://www.ema.europa.eu/en/medicines/human/EPAR/ovaleap.

[12] European Medicines Agency European Public Assessment Report (EPAR): Bemfola® (Follitropin Alfa). https://www.ema.europa.eu/en/medicines/human/EPAR/bemfola.

[13] European Medicines Agency Guideline on Similar Biological Medicinal Products Containing Biotechnology-Derived Proteins as Active Substance: Non-Clinical and Clinical Issues. 18 December 2014. https://www.ema.europa.eu/en/documents/scientific-guideline/guideline-similar-biological-medicinal-products-containing-biotechnology-derived-proteins-active_en-2.pdf.

[14] International Conference on Harmonisation. ICH Q5E Biotechnological/Biological Products Subject to Changes in Their Manufacturing Process: Comparability of Biotechnological/Biological Products. https://www.ema.europa.eu/en/documents/scientific-guideline/ich-q-5-e-comparability-biotechnological/biological-products-step-5_en.pdf.

[15] Hye-Na K., Thorpe R., Knezevica I. (2020). The regulatory landscape of biosimilars: WHO efforts and progress made from 2009 to 2019. Biologicals.

[16] Grampp G., Ramanan S. (2015). The Diversity of Biosimilar Design and Development: Implications for Policies and Stakeholders. BioDrugs.

[17] Mastrangeli R., Satwekar A., Cutillo F., Ciampolillo C., Palinsky W., Longobardi S. (2017). In-vivo biological activity and glycosylation analysis of a biosimilar recombinant human follicle-stimulating hormone product (Bemfola) compared with its reference medicinal product (GONAL-f). PLoS ONE.

[18] de Mora F., Fauser B.C.J.M. (2017). Biosimilars to recombinant human FSH medicines: Comparable efficacy and safety to the original biologic. Reprod. BioMed. Online.

[19] European Medicines Agency Guideline on Non-Clinical and Clinical Development of Similar Biological Medicinal Products Containing Recombinant Human Follicle Stimulating Hormone (r-hFSH). https://www.ema.europa.eu/en/documents/scientific-guideline/guideline-non-clinical-clinical-development-similar-biological-medicinal-products-containing_en.pdf.

[20] Rettenbacher M., Andersen A.N., Garcia-Velasco J.A., Sator M., Barri P., Lindenberg S., van der Ven K., Khalaf Y., Bentin-Ley U., Obruca A. (2015). A multi-centre phase 3 study comparing efficacy and safety of Bemfola^®^ versus Gonal-f^®^ in women undergoing ovarian stimulation for IVF. Reprod. BioMed. Online.

[21] Strowitzki T., Kuczynski W., Mueller A., Bias P. (2016). Randomized, active-controlled, comparative phase 3 efficacy and safety equivalence trial of Ovaleap^®^ (recombinant human follicle-stimulating hormone) in infertile women using assisted reproduction technology (ART). Reprod. Biol. Endocrinol..

[22] Braam S.C., de Bruin J.P., Buisman E., Brandes M., Nelen W., Smeenk J.M.J., van der Steeg J.W., Mol B.W.J., Hamilton C. (2018). Treatment strategies and cumulative live birth rates in WHO-II ovulation disorders. Eur. J. Obstet. Gynecol. Reprod. Biol..

[23] Germond M., Urner F., Chanson A., Primi M.P., Wirthner D., Senn A. (2004). What is the most relevant standard of success in assisted reproduction?: The cumulated singleton/twin delivery rates per oocyte pick-up: The CUSIDERA and CUTWIDERA. Hum. Reprod..

[24] Malizia B.A., Hacker M.R., Penzias A.S. (2009). Cumulative live-birth rates after in vitro fertilization. N. Engl. J. Med..

[25] Nayudu P.L., Vitt U.A., Barrios De Tomasi J., Pancharatna K., Ulloa-Aguirre A. (2002). Intact follicle culture: What it can tell us about the roles of FSH glycoforms during follicle development. Reprod. BioMed. Online.

[26] Selman H., Pacchiarotti A., El-Danasouri I. (2009). Ovarian stimulation protocols based on follicle-stimulating hormone glycosylation pattern: Impact on oocyte quality and clinical outcome. Fertil. Steril..

[27] Orvieto R., Seifer D.B. (2016). Biosimilar FSH preparations- are they identical twins or just siblings?. Reprod. Biol. Endocrinol..

[28] Chua S.J., Mol B.W., Longobardi S., Orvieto R., Venetis C.A., Lispi M., Storr A., D’Hooghe T. (2021). Biosimilar recombinant follitropin alfa preparations versus the reference product (Gonal-F(R)) in couples undergoing assisted reproductive technology treatment: A systematic review and meta-analysis. Reprod. Biol. Endocrinol..

[29] Andersen C., Ezcurra D. (2014). Human steroidogenesis: Implications for controlled ovarian stimulation with exogenous gonadotropins. Reprod. Biol. Endocrinol..

[30] Dias J.A., Ulloa-Aguirre A. (2021). New Human Follitropin Preparations: How Glycan Structural Differences May Affect Biochemical and Biological Function and Clinical Effect. Front. Endocrinol..

[31] Bousfield G.R., Butnev V.Y., White W.K., Hall A.S., Harvey D.J. (2015). Comparison of Follicle-Stimulating Hormone Glycosylation Microheterogenity by Quantitative Negative Mode Nano-Electrospray Mass Spectrometry of Peptide-N Glycanase-Released Oligosaccharides. J. Glycom. Lipidom..

[32] Wide L., Eriksson K. (2018). Low-glycosylated forms of both FSH and LH play major roles in the natural ovarian stimulation. Ups. J. Med. Sci..

[33] Zambrano E., Olivares A., Mendez J.P., Guerrero L., Díaz-Cueto L., Veldhuis J.D., Ulloa-Aguirre A. (1995). Dynamics of basal and gonadotropin-releasing hormone-releasable serum follicle-stimulating hormone charge isoform distribution throughout the human menstrual cycle. J. Clin. Endocrinol. Metab..

[34] Riccetti L., Sperduti S., Lazzaretti C., Klett D., De Pascali F., Paradiso E., Limoncella S., Potì F., Tagliavini S., Trenti T. (2019). Glycosylation Pattern and in vitro Bioactivity of Reference Follitropin alfa and Biosimilars. Front. Endocrinol..

[35] Ulloa-Aguirre A., Timossi C., Mendez J.P. (2001). Is there any physiological role for gonadotrophin oligosaccharide heterogeneity in humans? I. Gondatrophins are synthesized and released in multiple molecular forms. A matter of fact. Hum. Reprod..

[36] Bousfield G.R., Harvey D.J. (2019). Follicle-Stimulating Hormone Glycobiology. Endocrinology.

[37] Leão Rde B., Esteves S.C. (2014). Gonadotropin therapy in assisted reproduction: An evolutionary perspective from biologics to biotech. Clinics.

[38] Walsh G., Jefferis R. (2006). Post-translational modifications in the context of therapeutic proteins. Nat. Biotechnol..

[39] Zhu A., Hurst R. (2002). Anti-N-glycolylneuraminic acid antibodies identified in healthy human serum. Xenotransplantation.

[40] Visser E.A., Moons S.J., Timmermans S.B.P.E., de Jong H., Boltje T.J., Büll C. (2021). Sialic acid O-acetylation: From biosynthesis to roles in health and disease. J. Biol. Chem..

[41] Hermeling S., Crommelin D.J.A., Schellekens H., Jiskoot W. (2004). Structure-immunogenicity relationships of therapeutic proteins. Pharm. Res..

[42] Bassett R., Lispi M., Ceccarelli D., Grimaldi L., Mancinelli M., Martelli F., Van Dorsselaer A. (2009). Analytical identification of additional impurities in urinary-derived gonadotrophins. Reprod. BioMed. Online.

[43] Lispi M., Bassett R., Crisci C., Mancinelli M., Martelli F., Ceccarelli D., De Bellis C., Mendola D. (2006). Comparative assessment of the consistency and quality of a highly purified FSH extracted from human urine (urofollitropin) and a recombinant human FSH (follitropin α). Reprod. BioMed. Online.

[44] Meher B.R., Dixit A., Bousfield G.R., Lushington G.H. (2015). Glycosylation Effects on FSH-FSHR Interaction Dynamics: A Case Study of Different FSH Glycoforms by Molecular Dynamics Simulations. PLoS ONE.

[45] Jiang X., Dias J.A., He X. (2014). Structural biology of glycoprotein hormones and their receptors: Insights to signalling. Mol. Cell. Endocrinol..

[46] Barrios-De-Tomasi J., Timossi C., Merchant H., Quintanar A., Avalos J.M., Andersen C.Y., Ulloa-Aguirre A. (2002). Assessment of the in vitro and in vivo biological activities of the human follicle-stimulating isohormones. Mol. Cell. Endocrinol..

[47] Asian-Pacific Biotech News An Exclusive on LG Life Sciences. https://www.asiabiotech.com/12/1203/0022_0028.pdf.

[48] Barakhoeva Z., Vovk L., Fetisova Y., Marilova N., Ovchinnikova M., Tischenko M., Scherbatyuk Y., Kolotovkina A., Miskun A., Kasyanova G. (2019). A multicenter, randomized, phase III study comparing the efficacy and safety of follitropin alpha biosimilar and the original follitropin alpha. Eur. J. Obstet. Gynecol. Reprod. Biol..

[49] Drugs.com Follitrope. https://www.drugs.com/international/follitrope.html..

[50] Generics and Biosimilar Initiative. Folisurge Summary of Product Characteristics. https://www.gabionline.net/biosimilars/general/Similar-biologics-approved-and-marketed-in-India.

[51] Hu L., Zhang S., Quan S., Lv J., Qian W., Huang Y., Lu W., Sun Y. (2020). Efficacy and safety of recombinant human follicle-stimulating hormone in patients undergoing in vitro fertilization-embryo transfer. Aging.

[52] Medsintez Plant The First Russian Medicine for Infertility, Primapur® by Medsintez Plant, Will Reach Clinics in the New Year. http://www.medsintez.com/en/novosti/199-the-first-russian-medicine-for-infertility-primapur-by-medsintez-plant-will-reach-clinics-in-the-new-year.

[53] PR Newswire China Recombinant Human Follitropin Market Report 2018–2022. Featuring Merck Serono (GONAL-f) & GeneScience Pharmaceuticals Co., Ltd. (Jinsaiheng). https://www.prnewswire.com/news-releases/china-recombinant-human-follitropin-market-report-2018-2022-featuring-merck-serono-gonal-f-genescience-pharmaceuticals-co-ltd-jinsaiheng-300776822.html.

[54] Budani M.C., Fensore S., Di Marzio M., Tiboni G.M. (2020). Efficacy and safety of follitropin alpha biosimilars compared to their reference product: A meta-analysis. Gynecol. Endocrinol..

[55] Vitt U.A., Nayudu P.L., Rose U.M., Kloosterboer H.J. (2001). Embryonic development after follicle culture is influenced by follicle-stimulating hormone isoelectric point range. Biol. Reprod..

[56] Andersen C., Westergaard L., van Wely M. (2004). FSH isoform composition of commercial gonadotrophin preparations: A neglected aspect?. Reprod BioMed. Online.

[57] Heikinheimo O., Bitzer J., Garcia Rodriguez L. (2017). Real-world research and the role of observational data in the field of gynaecology—A practical review. Eur. J. Contracept. Reprod. Health Care.

[58] Benchaib M., Grynberg M., Cedrin-Durnerin I., Raguideau F., Lennon H., Castello-Bridoux C., Paillet S., Porte F., Verpillat P., Van Hille B. (2021). Effectiveness and treatment cost of assisted reproduction technology for women stimulated by gonadotropin in France: A cohort study using the National Health Database. Hum. Reprod..

[59] Iegemiddelverk S. Decision on Admission to the Exchange List for Follitropin Alfa. https://legemiddelverket.no/.

[60] Australian Government Department of Health Therapeutic Goods Administration. Australian Public Assessment Report for Follitropin Alfa. https://www.tga.gov.au/sites/default/files/auspar-follitropin-alfa-rch-160408.docx.

[61] Sinegubova M., Vorobiev I., Klishin A., Eremin D., Orlova N., Orlova N., Polzikov M. (2022). Purification Process of a Recombinant Human Follicle Stimulating Hormone Biosimilar (Primapur^®^) to Yield a Pharmaceutical Product with High Batch-to-Batch Consistency. Pharmaceutics.

[62] Pignataro M.F., Herrera M.G., DoderClick or tap here to enter text.o, V.I. (2020). Evaluation of Peptide/Protein Self-Assembly and Aggregation by Spectroscopic Methods. Molecules.

