# Supplementary material for: Comparative Assessment of the Structural Features of Originator Recombinant Human Follitropin Alfa Versus Recombinant Human Follitropin Alfa Biosimilar Preparations Approved in Non-European Regions
Source: Int J Mol Sci. 2022 Jun 17;23(12):6762. doi: 10.3390/ijms23126762 (PMC9223809; doi:10.3390/ijms23126762)
Supplement: Supplementary file 1 [file ijms-23-06762-s001.zip › ijms-1734183-supplementary/ijms-1734183-supplementary.pdf]

Supplementary Appendix

Supplementary Table S1: S-index and A-index values per tested

batch<br>Glycosylation site	Index	GONAL-F		Corneumon	Primapur		Jin Sai Heng	
		BA057111	BA064607	RFU19002	0011019A102	0010220B02	201812049	55201910044
Asn52 - α	S-Index	1.7	1.8	2.0	1.4	1.6	1.7	1.6
	A-Index	2.2	2.2	2.3	2.3	2.3	2.2	2.2
Asn78 - α	S-Index	1.7	1.8	2.0	1.4	1.6	1.8	1.8
	A-Index	2.2	2.2	2.3	2.3	2.3	2.2	2.2
Asn7 - β	S-Index	2.9	2.9	3.2	2.6	2.8	2.8	2.8
	A-Index	3.3	3.3	3.5	3.6	3.5	3.3	3.4
Asn24 - β	S-Index	1.9	1.9	2.2	1.7	1.8	1.9	1.9
	A-Index	2.2	2.2	2.4	2.3	2.3	2.2	2.2
Average	S-Index	2.1	2.1	2.4	1.8	1.9	2.0	2.0
	A-Index	2.5	2.5	2.6	2.6	2.6	2.5	2.5

Glycosylation site	Index	GONAL-f		Follitrope		Folisurge			
		BA057111	BA064607	RFV19009	RFV19005	7070091	7150015	7150014	7070093
Asn52 - α	S-Index	1.8	1.8	2.0	2.0	1.6	1.6	1.6	1.6
	A-Index	2.2	2.2	2.3	2.2	2.1	2.1	2.1	2.1
Asn78 - α	S-Index	1.7	1.7	2.0	2.0	1.7	1.7	1.7	1.7
	A-Index	2.2	2.1	2.2	2.2	2.1	2.1	2.1	2.1
Asn7 - β	S-Index	2.9	2.9	3.1	3.1	2.7	2.7	2.8	2.8
	A-Index	3.3	3.3	3.4	3.4	3.1	3.1	3.1	3.1
Asn24 - β	S-Index	1.9	1.9	2.2	2.2	1.9	1.9	1.9	1.9

Glycosylation site	Index	GONAL-f		Follitrope		Folisurge			
		BA057111	BA064607	RFV19009	RFV19005	7070091	7150015	7150014	7070093
	A-Index	2.2	2.2	2.4	2.4	2.1	2.1	2.2	2.1
Average	S-Index	2.1	2.1	2.3	2.3	2.0	2.0	2.0	2.0
	A-Index	2.5	2.4	2.6	2.6	2.4	2.4	2.4	2.4

Supplementary Table S2: Identity and relative abundance of post-translational modifications detected in r-hFSH-alfa preparations

Subunit	Post-translational modifications	Gonal-F		Follitrope LG		Folisurge			
		BA057111	BA064607	RFV19009	RFV19005	7070091	7150015	7150014	7070093
α	N-terminal heterogeneity (N-2)	8.5	4.9	16.6	9.8	6.4	18.8	18.0	15.2
	Oxidation Met 29	0.6	1.0	2.3	2.1	2.1	3.1	2.8	3.3
	Oxidation Met 71	N.D.	N.D.	2.2	3.2	N.D.	N.D.	N.D.	N.D.
β	N-terminal heterogeneity (N-2)	51.3	50.4	47.0	46.6	54.3	56.2	55.7	56.3
	Succinimide Asp 71	0.1	0.1	N.D.	N.D.	0.1	0.1	0.1	0.2
	Oxidation Met 109	0.8	0.8	1.3	2.0	1.7	1.6	2.0	1.6

Subunit	Post-translational modifications	Gonal-F		Corneumon	Primapur		Jin Sai Heng	
		BA057111	BA064607	RFU19002	0011019A102	0010220B02	201812049	55201910044
α	N-terminal heterogeneity (N-2)	7.2	4.9	6.6	10.4	14.3	5.0	5.0
	Oxidation Met 29	0.6	1.0	1.0	1.0	1.6	1.6	1.8
	Oxidation Met 71	N.D.	N.D.	N.D.	N.D.	N.D.	N.D.	N.D.
β	N-terminal heterogeneity (N-2)	48.6	51.4	46.1	57.0	51.8	48.8	47.7
	Succinimide Asp 71	N.D.	N.D.	0.2	N.D.	0.2	N.D.	N.D.
	Oxidation Met 109	0.7	0.8	1.5	0.5	0.5	0.6	0.7

Asp, aspartic acid; Met, methionine; N.D., not detected. The tables above detail the identity and location of post-translational modifications detected in the various preparations together with their relative abundance.
